# 9-(2-Chloro­benz­yloxy)-6,7-dihydro-2*H*-benzo[*c*][1,2,4]triazolo[4,3-*a*]azepin-3(5*H*)-one

**DOI:** 10.1107/S1600536811024470

**Published:** 2011-06-25

**Authors:** Da-Cheng Jin, Wen-Bin Zhang, Feng-Yu Piao, Rong-Bi Han

**Affiliations:** a Institute of Chemical Technology of Yanbian University, Yanji 133002, Jilin Province, People’s Republic of China; b Department of Chemistry, College of Science, Yanbian University, Yanji 133002, Jilin Province, People’s Republic of China; c Key Laboratory of Natural Resources of Changbai Mountain & Functional Molecules (Yanbian University), Ministry of Education, Yanji 133002, Jilin Province, People’s Republic of China

## Abstract

In the title mol­ecule, C_18_H_16_ClN_3_O_2_, the seven-membered ring adopts an envelope conformation with the flap atom deviating by 0.801 (5) Å from the mean plane formed by the remaining non-H atoms. Inter­molecular N—H⋯O hydrogen bonds link the mol­ecules into centrosymmetric dimers. The crystal packing also exhibits weak inter­molecular C—H⋯N hydrogen bonds and π–π inter­actions with a short distance of 3.734 (3) Å between the centroids of the aromatic rings of neighbouring mol­ecules.

## Related literature

For background and details of the synthesis, see: Piao *et al.* (2011 ▶); Jin *et al.* (2006 ▶). For related structures, see: Han *et al.* (2010 ▶); Jin *et al.* (2010 ▶).
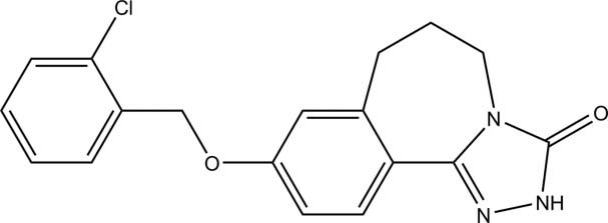


## Experimental

### 

#### Crystal data

C_18_H_16_ClN_3_O_2_
*M* *_r_* = 341.79
Monoclinic, 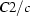

*a* = 28.421 (11) Å
*b* = 8.009 (4) Å
*c* = 14.896 (8) Å
β = 112.654 (18)°
*V* = 3129 (3) Å^3^
*Z* = 8
Mo *K*α radiation
μ = 0.26 mm^−1^
*T* = 291 K
0.35 × 0.28 × 0.25 mm

#### Data collection

Rigaku R-AXIS RAPID diffractometer
Absorption correction: multi-scan (*ABSCOR*; Higashi, 1995 ▶) *T*_min_ = 0.914, *T*_max_ = 0.938
14713 measured reflections
3569 independent reflections
3033 reflections with *I* > 2σ(*I*)
*R*_int_ = 0.019

#### Refinement

*R*[*F* ^2^ > 2σ(*F* ^2^)] = 0.040
*wR*(*F* ^2^) = 0.113
*S* = 1.07
3569 reflections
217 parameters
H-atom parameters constrained
Δρ_max_ = 0.21 e Å^−3^
Δρ_min_ = −0.36 e Å^−3^

### 

Data collection: *RAPID-AUTO* (Rigaku, 1998 ▶); cell refinement: *RAPID-AUTO*; data reduction: *CrystalStructure* (Rigaku/MSC, 2002 ▶); program(s) used to solve structure: *SHELXS97* (Sheldrick, 2008 ▶); program(s) used to refine structure: *SHELXL97* (Sheldrick, 2008 ▶); molecular graphics: *PLATON* (Spek, 2009 ▶); software used to prepare material for publication: *SHELXL97*.

## Supplementary Material

Crystal structure: contains datablock(s) global, I. DOI: 10.1107/S1600536811024470/cv5117sup1.cif

Structure factors: contains datablock(s) I. DOI: 10.1107/S1600536811024470/cv5117Isup2.hkl

Supplementary material file. DOI: 10.1107/S1600536811024470/cv5117Isup3.cml

Additional supplementary materials:  crystallographic information; 3D view; checkCIF report

## Figures and Tables

**Table 1:** Hydrogen-bond geometry (Å, °)

*D*—H⋯*A*	*D*—H	H⋯*A*	*D*⋯*A*	*D*—H⋯*A*
N3—H3*A*⋯O2^i^	0.86	1.95	2.7986 (17)	170
C15—H15*B*⋯N2^ii^	0.97	2.66	3.410 (2)	134

Symmetry codes: (i) 
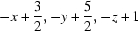
; (ii) 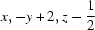.

**Fig. 1.**
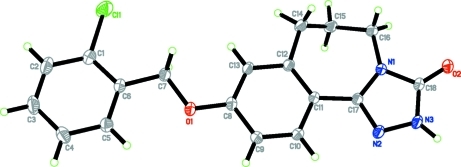
The molecular structure of (I) with the atom numbering. Displacement ellipsoids are drawn at the 30% probalility level.

## supplementary crystallographic information

### Comment

The title compound (I) was obtained from the reaction of 7-alkoxy-2,3,4,5-tetrahydro-1*H*-benzo[*c*]azepin-1-thione and methyl hydrazinocarboylate in n-butanol (Piao *et al.* 2011; Jin *et al.* 2006), but its structure can not be confirmed with ^13^C-NMR spectra. Herein we report its crystal structure.

In (I) (Fig. 1), all bond lengths and angles are normal and correspond to those reported for the related compounds (Han *et al.* 2010; Jin *et al.* 2010). Except C15, all  non-hydrogen atoms lie in a plane with r.m.s of 0.0406 (9) Å. Intermolecular N—H···O hydrogen bonds (Table 1) link the molecules into centrosymmetric dimers. The crystal packing exhibits weak intermolecular C—H···N hydrogen bonds (Table 1) and π—π interactions with the short distence of 3.734 (3) Å between the centroids of aromatic rings from the neighbouring molecules.

### Experimental

The title compound was prepared according to the literature (Piao *et al.*, 2011; Jin *et al.*, 2006). Single crystals suitable for X-ray diffraction were prepared by slow evaporation a mixture of n-butanol and ethanol (1:1) at room temperature.

### Refinement

C-bound H atoms were placed in calculated positions (C—H 0.93-0.97 Å) and were included in the refinement in the riding model, with *U*_iso_(H) = 1.2 *U*_eq_(C). The H atom bound to N3 was placed in the calculated position with N—H = 0.86 Å and refined with *U*_iso_(H) = 1.2 *U*_eq_(N).

### Crystal data

**Table:**

C_18_H_16_ClN_3_O_2_	*F*(000) = 1424
*M**_r_* = 341.79	*D*_x_ = 1.451 Mg m^−^^3^
Monoclinic, *C*2/*c*	Mo *K*α radiation, λ = 0.71073 Å
Hall symbol: -C 2yc	Cell parameters from 12565 reflections
*a* = 28.421 (11) Å	θ = 3.1–27.6°
*b* = 8.009 (4) Å	µ = 0.26 mm^−^^1^
*c* = 14.896 (8) Å	*T* = 291 K
β = 112.654 (18)°	Block, colourless
*V* = 3129 (3) Å^3^	0.35 × 0.28 × 0.25 mm
*Z* = 8	

### Data collection

**Table:**

Rigaku R-AXIS RAPID diffractometer	3569 independent reflections
Radiation source: fine-focus sealed tube	3033 reflections with *I* > 2σ(*I*)
graphite	*R*_int_ = 0.019
ω scans	θ_max_ = 27.5°, θ_min_ = 3.1°
Absorption correction: multi-scan (*ABSCOR*; Higashi, 1995)	*h* = −35→36
*T*_min_ = 0.914, *T*_max_ = 0.938	*k* = −8→10
14713 measured reflections	*l* = −19→19

### Refinement

**Table:**

Refinement on *F*^2^	Primary atom site location: structure-invariant direct methods
Least-squares matrix: full	Secondary atom site location: difference Fourier map
*R*[*F*^2^ > 2σ(*F*^2^)] = 0.040	Hydrogen site location: inferred from neighbouring sites
*wR*(*F*^2^) = 0.113	H-atom parameters constrained
*S* = 1.07	*w* = 1/[σ^2^(*F*_o_^2^) + (0.0667*P*)^2^ + 1.2865*P*] where *P* = (*F*_o_^2^ + 2*F*_c_^2^)/3
3569 reflections	(Δ/σ)_max_ = 0.002
217 parameters	Δρ_max_ = 0.21 e Å^−^^3^
0 restraints	Δρ_min_ = −0.36 e Å^−^^3^

### Special details

**Table:**

Experimental. (See detailed section in the paper)
Geometry. All e.s.d.'s (except the e.s.d. in the dihedral angle between two l.s. planes) are estimated using the full covariance matrix. The cell e.s.d.'s are taken into account individually in the estimation of e.s.d.'s in distances, angles and torsion angles; correlations between e.s.d.'s in cell parameters are only used when they are defined by crystal symmetry. An approximate (isotropic) treatment of cell e.s.d.'s is used for estimating e.s.d.'s involving l.s. planes.
Refinement. Refinement of *F*^2^ against ALL reflections. The weighted *R*-factor *wR* and goodness of fit *S* are based on *F*^2^, conventional *R*-factors *R* are based on *F*, with *F* set to zero for negative *F*^2^. The threshold expression of *F*^2^ > σ(*F*^2^) is used only for calculating *R*-factors(gt) *etc*. and is not relevant to the choice of reflections for refinement. *R*-factors based on *F*^2^ are statistically about twice as large as those based on *F*, and *R*- factors based on ALL data will be even larger.

### Fractional atomic coordinates and isotropic or equivalent isotropic displacement parameters (Å^2^)

**Table:**

	*x*	*y*	*z*	*U*_iso_*/*U*_eq_	
C1	1.12595 (5)	0.48226 (18)	0.42519 (11)	0.0357 (3)	
C2	1.17285 (6)	0.4037 (2)	0.45532 (13)	0.0466 (4)	
H2	1.1863	0.3715	0.4102	0.056*	
C3	1.19933 (6)	0.3737 (2)	0.55289 (14)	0.0499 (4)	
H3	1.2310	0.3217	0.5740	0.060*	
C4	1.17895 (5)	0.4207 (2)	0.61950 (12)	0.0441 (4)	
H4	1.1965	0.3982	0.6853	0.053*	
C5	1.13222 (5)	0.50153 (17)	0.58801 (11)	0.0348 (3)	
H5	1.1189	0.5342	0.6334	0.042*	
C6	1.10491 (5)	0.53472 (15)	0.49043 (10)	0.0285 (3)	
C7	1.05452 (5)	0.62455 (16)	0.45363 (9)	0.0302 (3)	
H7A	1.0568	0.7245	0.4188	0.036*	
H7B	1.0283	0.5531	0.4091	0.036*	
C8	0.99692 (4)	0.75568 (15)	0.51182 (9)	0.0280 (3)	
C9	0.98445 (5)	0.80010 (17)	0.58989 (9)	0.0321 (3)	
H9	1.0058	0.7714	0.6530	0.038*	
C10	0.94008 (5)	0.88719 (17)	0.57312 (9)	0.0305 (3)	
H10	0.9319	0.9164	0.6257	0.037*	
C11	0.90681 (4)	0.93338 (15)	0.47889 (9)	0.0256 (2)	
C12	0.91964 (5)	0.88864 (16)	0.40076 (9)	0.0283 (3)	
C13	0.96483 (5)	0.79982 (17)	0.41914 (9)	0.0300 (3)	
H13	0.9734	0.7697	0.3671	0.036*	
C14	0.88795 (6)	0.9227 (2)	0.29509 (10)	0.0419 (4)	
H14A	0.8630	0.8338	0.2711	0.050*	
H14B	0.9101	0.9166	0.2594	0.050*	
C15	0.85993 (5)	1.08733 (19)	0.27128 (10)	0.0378 (3)	
H15A	0.8820	1.1745	0.3106	0.045*	
H15B	0.8521	1.1141	0.2035	0.045*	
C16	0.81139 (6)	1.0853 (2)	0.28893 (10)	0.0455 (4)	
H16A	0.7919	1.1848	0.2605	0.055*	
H16B	0.7913	0.9893	0.2563	0.055*	
C17	0.86043 (4)	1.02428 (15)	0.47298 (9)	0.0261 (2)	
C18	0.78474 (5)	1.15106 (18)	0.42277 (10)	0.0325 (3)	
Cl1	1.091807 (19)	0.51334 (6)	0.30151 (3)	0.05919 (16)	
N1	0.81980 (4)	1.07857 (14)	0.39169 (8)	0.0305 (2)	
N2	0.85271 (4)	1.05951 (16)	0.55146 (8)	0.0359 (3)	
N3	0.80577 (4)	1.13648 (16)	0.51955 (9)	0.0377 (3)	
H3A	0.7916	1.1713	0.5577	0.045*	
O1	1.04149 (3)	0.66817 (13)	0.53366 (7)	0.0348 (2)	
O2	0.74323 (4)	1.21405 (16)	0.37020 (7)	0.0452 (3)	

### Atomic displacement parameters (Å^2^)

**Table:**

	*U*^11^	*U*^22^	*U*^33^	*U*^12^	*U*^13^	*U*^23^
C1	0.0366 (7)	0.0354 (7)	0.0399 (7)	0.0023 (5)	0.0201 (6)	−0.0008 (6)
C2	0.0399 (8)	0.0489 (9)	0.0607 (10)	0.0052 (6)	0.0300 (7)	−0.0076 (7)
C3	0.0278 (7)	0.0536 (9)	0.0658 (11)	0.0099 (6)	0.0153 (7)	−0.0039 (8)
C4	0.0295 (7)	0.0483 (9)	0.0469 (8)	0.0033 (6)	0.0065 (6)	0.0015 (7)
C5	0.0297 (6)	0.0379 (7)	0.0374 (7)	0.0015 (5)	0.0136 (5)	−0.0019 (5)
C6	0.0268 (6)	0.0254 (6)	0.0361 (7)	0.0002 (4)	0.0152 (5)	−0.0015 (5)
C7	0.0297 (6)	0.0309 (6)	0.0319 (6)	0.0050 (5)	0.0140 (5)	0.0004 (5)
C8	0.0246 (6)	0.0266 (6)	0.0338 (6)	0.0033 (4)	0.0124 (5)	−0.0002 (5)
C9	0.0298 (6)	0.0386 (7)	0.0265 (6)	0.0061 (5)	0.0094 (5)	0.0021 (5)
C10	0.0302 (6)	0.0379 (7)	0.0259 (6)	0.0049 (5)	0.0136 (5)	−0.0007 (5)
C11	0.0251 (6)	0.0261 (6)	0.0271 (6)	0.0012 (4)	0.0119 (4)	−0.0007 (4)
C12	0.0288 (6)	0.0311 (6)	0.0268 (6)	0.0047 (5)	0.0127 (5)	0.0017 (5)
C13	0.0310 (6)	0.0338 (7)	0.0294 (6)	0.0057 (5)	0.0163 (5)	−0.0007 (5)
C14	0.0463 (8)	0.0545 (9)	0.0259 (7)	0.0207 (7)	0.0149 (6)	0.0019 (6)
C15	0.0440 (8)	0.0460 (8)	0.0253 (6)	0.0127 (6)	0.0154 (5)	0.0060 (5)
C16	0.0358 (7)	0.0739 (11)	0.0235 (6)	0.0178 (7)	0.0077 (5)	−0.0028 (6)
C17	0.0257 (6)	0.0277 (6)	0.0264 (6)	0.0021 (4)	0.0119 (4)	0.0002 (4)
C18	0.0277 (6)	0.0399 (7)	0.0325 (6)	0.0050 (5)	0.0145 (5)	−0.0021 (5)
Cl1	0.0724 (3)	0.0747 (3)	0.0384 (2)	0.0194 (2)	0.0300 (2)	0.00614 (19)
N1	0.0249 (5)	0.0410 (6)	0.0260 (5)	0.0065 (4)	0.0101 (4)	−0.0024 (4)
N2	0.0334 (6)	0.0482 (7)	0.0292 (6)	0.0144 (5)	0.0156 (5)	0.0031 (5)
N3	0.0325 (6)	0.0529 (7)	0.0325 (6)	0.0155 (5)	0.0179 (5)	0.0019 (5)
O1	0.0293 (5)	0.0439 (6)	0.0324 (5)	0.0127 (4)	0.0133 (4)	0.0010 (4)
O2	0.0291 (5)	0.0698 (7)	0.0355 (5)	0.0178 (5)	0.0113 (4)	−0.0014 (5)

### Geometric parameters (Å, °)

**Table:**

C1—C2	1.383 (2)	C11—C12	1.3938 (18)
C1—C6	1.3879 (19)	C11—C17	1.4785 (16)
C1—Cl1	1.7379 (18)	C12—C13	1.4000 (17)
C2—C3	1.376 (3)	C12—C14	1.5071 (19)
C2—H2	0.9300	C13—H13	0.9300
C3—C4	1.380 (2)	C14—C15	1.510 (2)
C3—H3	0.9300	C14—H14A	0.9700
C4—C5	1.387 (2)	C14—H14B	0.9700
C4—H4	0.9300	C15—C16	1.501 (2)
C5—C6	1.384 (2)	C15—H15A	0.9700
C5—H5	0.9300	C15—H15B	0.9700
C6—C7	1.5048 (17)	C16—N1	1.4565 (19)
C7—O1	1.4214 (17)	C16—H16A	0.9700
C7—H7A	0.9700	C16—H16B	0.9700
C7—H7B	0.9700	C17—N2	1.3008 (18)
C8—O1	1.3726 (15)	C17—N1	1.3827 (16)
C8—C13	1.3759 (19)	C18—O2	1.2441 (16)
C8—C9	1.3865 (19)	C18—N3	1.3363 (19)
C9—C10	1.3770 (18)	C18—N1	1.3782 (16)
C9—H9	0.9300	N2—N3	1.3774 (15)
C10—C11	1.4066 (18)	N3—H3A	0.8600
C10—H10	0.9300		
			
C2—C1—C6	122.12 (14)	C11—C12—C14	125.62 (11)
C2—C1—Cl1	118.94 (12)	C13—C12—C14	115.49 (11)
C6—C1—Cl1	118.93 (11)	C8—C13—C12	122.04 (11)
C3—C2—C1	119.25 (14)	C8—C13—H13	119.0
C3—C2—H2	120.4	C12—C13—H13	119.0
C1—C2—H2	120.4	C12—C14—C15	116.89 (12)
C2—C3—C4	120.11 (14)	C12—C14—H14A	108.1
C2—C3—H3	119.9	C15—C14—H14A	108.1
C4—C3—H3	119.9	C12—C14—H14B	108.1
C3—C4—C5	119.77 (15)	C15—C14—H14B	108.1
C3—C4—H4	120.1	H14A—C14—H14B	107.3
C5—C4—H4	120.1	C16—C15—C14	112.75 (14)
C6—C5—C4	121.43 (14)	C16—C15—H15A	109.0
C6—C5—H5	119.3	C14—C15—H15A	109.0
C4—C5—H5	119.3	C16—C15—H15B	109.0
C5—C6—C1	117.29 (12)	C14—C15—H15B	109.0
C5—C6—C7	122.98 (11)	H15A—C15—H15B	107.8
C1—C6—C7	119.72 (12)	N1—C16—C15	113.26 (11)
O1—C7—C6	109.31 (11)	N1—C16—H16A	108.9
O1—C7—H7A	109.8	C15—C16—H16A	108.9
C6—C7—H7A	109.8	N1—C16—H16B	108.9
O1—C7—H7B	109.8	C15—C16—H16B	108.9
C6—C7—H7B	109.8	H16A—C16—H16B	107.7
H7A—C7—H7B	108.3	N2—C17—N1	110.23 (11)
O1—C8—C13	124.30 (11)	N2—C17—C11	120.56 (11)
O1—C8—C9	116.29 (11)	N1—C17—C11	129.18 (11)
C13—C8—C9	119.40 (12)	O2—C18—N3	129.51 (12)
C10—C9—C8	119.33 (12)	O2—C18—N1	126.32 (13)
C10—C9—H9	120.3	N3—C18—N1	104.16 (11)
C8—C9—H9	120.3	C18—N1—C17	107.84 (11)
C9—C10—C11	122.07 (12)	C18—N1—C16	119.35 (11)
C9—C10—H10	119.0	C17—N1—C16	132.51 (11)
C11—C10—H10	119.0	C17—N2—N3	105.25 (11)
C12—C11—C10	118.30 (11)	C18—N3—N2	112.51 (10)
C12—C11—C17	126.21 (11)	C18—N3—H3A	123.7
C10—C11—C17	115.48 (11)	N2—N3—H3A	123.7
C11—C12—C13	118.86 (11)	C8—O1—C7	116.19 (10)

### Hydrogen-bond geometry (Å, °)

**Table:**

*D*—H···*A*	*D*—H	H···*A*	*D*···*A*	*D*—H···*A*
N3—H3A···O2^i^	0.86	1.95	2.7986 (17)	170
C15—H15B···N2^ii^	0.97	2.66	3.410 (2)	134

Symmetry codes: (i) −*x*+3/2, −*y*+5/2, −*z*+1; (ii) *x*, −*y*+2, *z*−1/2.
